# The Quality of Life (QOL) after Total Knee Arthroplasties among Saudi Arabians: A Pilot Study

**Published:** 2014-09

**Authors:** Abdallah S. Al-Omran

**Affiliations:** Department of Orthopaedic Surgery, College of Medicine, University of Dammam, King Fahd Hospital of the University, AlKhobar, Saudi Arabia

**Keywords:** Total knee arthroplasty, Saudi Arabians, quality of life

## Abstract

**Background and Objective:**

Total knee arthroplasty (TKA) is commonly performed in Saudi Arabia but there is very limited published data on outcome and quality of life (QOL) post Knee arthroplasty. To assess the QOL post TKA we performed this retrospective study.

**Methods:**

Total Knee arthroplasty was started in mid- 2000’s at the university hospital. Fifty–two patients of TKA who came for follow up during the study period were asked to fill a pre-determined questionnaire and clinical examination, were included in the study. Patients were assessed and at 2 parameters pre and postoperative time-points, for pain [1-9], walking [1-9] and asked whether they would recommend the procedure to their relatives and friends, and finally whether they were satisfied with the outcome.

**Results:**

We interviewed 52 patients (9 males and 43 females), mean age of 64.75 ± 7.90 years. Twenty (34.50%) had bilateral TKR, and the rest single sided. The preoperative night pain was 7.72 ± 2.03 compared to postoperative 1.92 ± 1.41 (*P*<0.001 (5.80 and < 6.47) and pain at walking was 8.39 ± 0.77 versus 2.39 ± 2.05 (*P*<0.001(5.40 and < 6.55). The overall satisfaction 93% (8.37 ± 1.32) and QOL as assessed preoperatively was 3.60 ± 2.15 and postoperatively was 8.41  ±  1.27 (*P*<0.001 (4.81and 4.13). Fifty-one (98.07%) patients indicated that they will recommend the procedure to others.

**Conclusions:**

The overall satisfaction and improvement of QOL in male patients was 93.77% and female patients 92.77% and all patients indicated that they will recommend others to undergo the similar procedure to improve their QOL.

## INTRODUCTION

Osteoarthritis (OA) of the knee is a common cause of pain and disability in the aging population of the world (1). The incidence of OA among Saudi Arabians is reported to be 13%, but it can reach between 30-60% in selected regions of country (2). Conservative therapy in OA takes from comprises of non-steriodal anti inflammatory drugs, physical therapy, modification of life style and nutritional supplements (3-5). These modalities may just postpone the final surgical interference by few years. During this period of time pain due to OA decrease the physical activity in patients with a real negatively impact on the QOl (6, 7). To improve the QOL many strides took place since 1969, the first TKA, in the design and longevity of the implants. The QOL in general is described as limitations in daily essential activities which could further impact the health of the older adults and intervention could improve such health status (8). Kyo *et al*. (9) reported that patients after joint replacement had significant improvements in health related quality of life.

Currently TKR is the most common surgical treatment carried out standard of care for advanced OA (10-13). Recently Waimann *et al.* (14) concluded that TKR is an effective surgical intervention which reduces pain and improving functional status among patients with knee OA. In Saudi Arabia over two decades TKR is being done in many institutions but a search of English language literature including, Excerpta Medica, Cochrane reviews, DOAJ, EBSCO Publishing's Electronic Databases, Genamics JournalSeek, Global Health, Google Scholar, Health and Wellness Research Center, Journal Citation Reports, ProQuest, PubMed, Pubmed Central, Science Citation Index Directory and Web of Science and archives of Saudi Medical Journal and Annals of Saudi medicine, did not shows us any publication of assessment/improvement in the QOL of patients with OA of knee who underwent TKA. The principle is assessing the ideal management of any disease is to gauge the QOL at the end of the treatment. Total knee arthroplasty was reported to improve the QOL, but to this date the QOL post TKA was not assessed. The objective of this pilot study was to assess the QOL, improvement of daily activities and satisfaction among Saudi Arabian men and women who had TKA for OA.

## PATIENTS AND METHODS

Total Knee arthroplasty was started in mid- 2000’s a tertiary care university hospital TKA is being performed routinely in our tertiary care University Hospital. Institutional review board approval obtained and the study was conducted between June and September 2013, Al-Khobar, Saudi Arabia. Patients who came for the follow up during the study period were included in the study after they signed the consent for answering the questionnaire. During the study period 52 patients completed a pre-determined questionnaire and clinical examination. As it was a pilot study the parameters assessed had to be customized. Patients were explained that they should truthfully answer the questions without bias and in no way will affect the further treatment and follow up. The principal investigator was assigned to interview all the patients and the questions were explained in the local language. Patients were assessed and at 2 pre and postoperative time-points, for pain [1-9], walking [1-9] and whether they will undergo the second side if needed and recommend the procedure to their relatives and friends and finally whether they were satisfied with the outcome. The assessment of QOL after TKA is based on the pain and walking pre-operative and post operatively and is the most reliable and valid parameters (15). With QOL the maximum score each patient assessed is between 1 to 9. A score of 9 indicated that QOL improved by 100%.

The data was analyzed using SPSS (Statistical Package for the Social Sciences), version 14.0, Chicago, Illinois. Data is expressed as mean ± standard deviation (SD). Statistical significance differences between groups were determined with Student’s *t*-test and *p* values of <0.05 using 95% Confidence Interval (CI) were considered as significant.

## RESULTS

We interviewed 52 patients (9 males and 43 females, mean age of 64.75 ± 7.90 years) who underwent TKA. Twenty (34.50%) had bilateral TKR, 16 right side and the rest were left sided TKR. Table 1 gives the comparison between all the patients for pre and post-operative assessment. The preoperative night pain was 7.72 ± 2.03 (range 2-9) compared to post-operative 1.92 ± 1.41 (range 1-5) (*P*<0.001 (-5.80 and -6.47) and pain at walking was 8.39 ± 0.77 (Range 7-9) versus 2.39 ± 2.05(Range 1-5) (*P*<0.001 (-5.40 and -6.55).

**Table 1:** Comparison of the data between Preoperative and Post Operative

Parameters	Before TKA (Range)	After TKA (Range)	*P* Value	(95% CI of difference) Lower Upper Bound Bound	

Pain at Night	7.72 ± 2.03 (2-9)	1.92 ± 1.41 (1-5)	<0.001	-5.80	-6.47
Pain at Walking	8.39 ± 0.77 (7-9)	2.39 ± 2.05 (1-5)	<0.001	-5.40	-6.55
QOL	3.60 ± 2.15 (1-5)	8.41 ± 1.27 (6-9)	<0.001	-4.81	-4.13

The overall satisfaction 93% (8.37 ± 1.32) and QOL as assessed preoperatively was 3.60 ± 2.15 and postoperatively was 8.41 ± 1.27 (*P*<0.001 (-4.81and -4.13).

Table 2 is the comparison between the male and female patients. Males were few in number but significantly older than female patients (72 ± 8.48 versus 63.3 ± 7 years *P*<0.005 (95% CI at -8.7 and -13.27).

**Table 2:** Data of Male and Female patients

Parameter	Male Patients	Female Patients	*P* Value	(95% CI of difference) Upper Lower Bound Bound	

Age	72 ± 8.48 (60-83)	63.30 ± 70 (55-81)	<0.005	-8.7	-13.27
Preoperative Night Pain	7.66 ± 1.93 (3-9)	7.73 ± 20 (2-9)	0.90	1.41	1.27
Pain on walking	8.44 ± 0.72 (7-9)	8.38 ± 0.79 (7-9)	0.82	0.44	0.57
Post Op Night Pain	2 ± 1.73 (1-5)	1.9 ± 1.35 (1-4)	0.82	1.04	1.24
Post-operative Pain on walking	2.24 ± 2.78 (1-6)	2.38 ± 1.89 (1-6)	0.82	1.13	1.09
Overall Satisfaction and QOL Improvement	8.44 ± 1.10 (7-9) (93.77%)	8.35 ± 1.37 (4-9) (92.77%)	0.84	0.71	0.98
Recommendation to others (Percent)	100	98			

Non-parametric test Wilcox Signed Rank Test was conducted with Bonferroni correction between the preoperative and post-operative pain scores which showed Z=-5.68 and *p*=0.0001, while pain on walking pre-operative and post-operative walking was Z=-5.71 with *p*=0.0001.

Fifty-one (98.07%) patients indicated that they will recommend the procedure to family and friends.

## DISCUSSION

Our study demonstrated that the QOL was significantly improved in patients who underwent TKA for osteoarthritis of the knee with over all satisfaction was over 93%. Secondly there was no gender differences in any of the pre and post-operative outcome parameters except that males were older than the females. Reports in the literature cite that there are significant differences among male and female patients with regard to the preoperative differences between the different assessment scores (15-17). Papakostidou *et al.* (18) found in their patients significant improvements in the parameters and QOL but there was gender differences in the improvement which is not borne by our study. Gonzalez *et al*. in a prospective study including a substantial number of patients found that Patients with higher baseline expectations for TJR, improved more in health related QOL at one year and had more likelihood to be satisfied than patients with lower expectations (19), while Mencière ML and his collegues concluded that QOL of operated TKA is better in patients who underwent regular design than in high flexed knee (20). Furthermore, a recent report found that Despite patient-reported improvements in pain, function, and physical activity after arthroplasty, objectively measured improvements in physical activity may not occur (21).

Our pilot study has shown that there was significant improvement in the QOL but this should be substantiated by studies with larger number of patients in the future.

The improvement of the pain and QOL post TKA has made many people suffering with advanced OA to undergo TKR, but still the acceptance of surgery differs from place to place. There is startling difference of acceptance by patients for TKR in US to United Kingdom of 10:25 (22, 23). Behairy, Motuweh, Kathlan (24) found that 67% in their study refused surgery for reasons like, being afraid of failed surgery, had misconception that TKR is too dangerous and some cited disability and death due to surgery itself. The findings of our study are important as patients need to be educated that TKA benefits and apprehension of such procedures are unfounded. In our study we found that the majority of the patients we assessed were females, even though the literature suggests that female patients underutilize joint replacement when compared to male patients (25). This is not true for the Saudi Arabian society as we observe more female patients with OA rather than males, hence they come in for TKA than males.

Osteoarthritis of the knee is a progressive degenerative disease due to normal ageing process and presents differently in different ethnic populations. Genetic influence, lifestyle and obesity which affects severity of the disease; this may ultimately affect the QOL assessment for comparison purposes (26). Added to these parameters, other studies post TKA suggested that even pre-intervention QOL scores, social support, comorbidities and the status of the mental health also influences the outcome (27, 28).

There is no doubt that our study has few limitations. The first being small number of patients interviewed and secondly we did not perform the scoring using international scoring system, but developed our own and with low number of male patients could not conclusively compare gender differences. The strengths of our study is that this is first such study anyone one has entertained in the Kingdom. Secondly, this pilot study suggests that we need to perform QOL scores on all the operated patients.

In conclusion we can say that TKA is rewarding for the surgeon and satisfying for the patients as they improve their QOL and live comfortably without pain and physical activity. Our study being first in Saudi Arabia, we believe other studies need to be performed at other centers which perform TKA so that we can recommend and educate patients as well as arthroplasty surgeons that the QOL can be improved post TKR making elderly patients mobile and prevent other complications due to immobility.
